# AMP-Activated Kinase Restricts Rift Valley Fever Virus Infection by Inhibiting Fatty Acid Synthesis

**DOI:** 10.1371/journal.ppat.1002661

**Published:** 2012-04-19

**Authors:** Theresa S. Moser, Daniel Schieffer, Sara Cherry

**Affiliations:** Department of Microbiology, Penn Genome Frontiers Institute, The University of Pennsylvania School of Medicine, Philadelphia, Pennsylvania, United States of America; University of Queensland, Australia

## Abstract

The cell intrinsic innate immune responses provide a first line of defense against viral infection, and often function by targeting cellular pathways usurped by the virus during infection. In particular, many viruses manipulate cellular lipids to form complex structures required for viral replication, many of which are dependent on *de novo* fatty acid synthesis. We found that the energy regulator AMPK, which potently inhibits fatty acid synthesis, restricts infection of the Bunyavirus, Rift Valley Fever Virus (RVFV), an important re-emerging arthropod-borne human pathogen for which there are no effective vaccines or therapeutics. We show restriction of RVFV both by AMPK and its upstream activator LKB1, indicating an antiviral role for this signaling pathway. Furthermore, we found that AMPK is activated during RVFV infection, leading to the phosphorylation and inhibition of acetyl-CoA carboxylase, the first rate-limiting enzyme in fatty acid synthesis. Activating AMPK pharmacologically both restricted infection and reduced lipid levels. This restriction could be bypassed by treatment with the fatty acid palmitate, demonstrating that AMPK restricts RVFV infection through its inhibition of fatty acid biosynthesis. Lastly, we found that this pathway plays a broad role in antiviral defense since additional viruses from disparate families were also restricted by AMPK and LKB1. Therefore, AMPK is an important component of the cell intrinsic immune response that restricts infection through a novel mechanism involving the inhibition of fatty acid metabolism.

## Author Summary

RNA viruses represent an important worldwide source of infection and disease in both humans and animals. While individual viruses have unique characteristics, some stages of infection are conserved and must be completed in order to successfully infect and grow. Viruses must undergo genome replication, protein synthesis, and assembly of new virus particles. In particular, numerous RNA viruses manipulate cellular membranes to create new complex structures required for viral replication in a process that is often dependent on fatty acid biosynthesis. This is a process that is tightly regulated by the energy sensor AMPK. We have found that energy-mediated activation of AMPK restricts infection of the Bunyavirus Rift Valley fever virus by decreasing levels of fatty acid synthesis. Furthermore, several additional RNA viruses from disparate families that share this dependence of membrane modification and fatty acid synthesis are also restricted by AMPK. Thus AMPK likely represents a novel component of the cell intrinsic immune response to RNA viruses, and may be a good target for the development of antiviral therapeutics against a range of medically important viruses.

## Introduction

Emerging and re-emerging arthropod-borne viral pathogens have lead to significant world-wide morbidity and mortality in humans and domestic animals, and are of medical and agricultural concern. Bunyaviruses are an important group of insect-borne RNA viruses that include disease causing members such as Sin Nombre, Hantavirus, Crimean-Congo hemorrhagic fever virus, and Rift Valley Fever Virus (RVFV). RVFV is a mosquito borne Category A agent initially endemic to sub-Saharan Africa. However, outbreaks of RVFV have recently occurred in Egypt and the Arabian Peninsula, indicating the potential of this virus to spread to new geographical areas [1]. RVFV has particular importance as an agricultural pathogen, where infection of livestock can lead to significant morbidity and mortality among young animals, and cause catastrophic abortion rates [1]. Most humans infected with RVFV develop self-limited febrile illness, although approximately 1–3% die from the disease due to hemorrhagic symptoms [2]–[5]. No effective vaccines or antiviral therapies have yet been developed against RVFV.

All viruses undergo sequential steps to complete their replication cycles. Bunyaviruses and other RNA viruses compartmentalize their RNA replication machinery on cellular membranes. An essential feature of these infections is the ability of viruses to rearrange and proliferate internal cellular membranes into distinct structures compartmentalizing the viral replication complex and supporting viral genome replication [6]. Depending on the virus, these membrane modifications can be derived from distinct cellular sources, including ER, Golgi, endosomal, and mitochondrial membranes, and may have complex biogenesis pathways derived from multiple intracellular origins [7]–[14]. Bunyamwera virus, a member of the Bunyavirus family related to RVFV, induces the formation of a new Golgi membrane-derived tubular structure with a globular head that harbors the viral replication complex [14], [15]. Disrupting the formation of this structure is associated with decreased levels of virus replication [15]. While different families of viruses use membranes derived from different cellular sources, and create membranous structures with distinct morphologies, there are some similarities in these structures, suggesting that commonalities exist in the mechanisms by which disparate viruses depend upon lipid metabolism or trafficking [16]. One clear point of overlap includes a requirement for cellular lipid biogenesis pathways and the generation of newly synthesized lipids [6]. Furthermore, enveloped viruses, which include Bunyaviruses, require incorporation of cellular membranes into their lipid envelopes during virus assembly, in a process that may also involve lipid modifications [17].

AMP-activated Kinase (AMPK) is a heterotrimeric complex that is the core energy sensor of the cells [18]. Thus AMPK activity is important for survival during periods of stress, and also has implications in type II diabetes, obesity, metabolic syndrome, longevity, and cancer [19]–[25]. The AMPK complex consists of a catalytic alpha subunit, and regulatory beta and gamma subunits [26]. Activation is triggered through binding of AMP or ADP to the Bateman domains of the gamma subunit, leading to increased phosphorylation of the threonine 172 on the alpha subunit by inducing allosteric activation and inhibiting dephosphorylation [27]–[30]. The canonical upstream activator that catalyzes this phosphorylation event is the constitutively active tumor suppressor LKB1, but additional activators such as CaMKKβ have been identified [31]–[35]. Under conditions of energetic stress, AMPK signals the cell to stop anabolic pathways and activate a catabolic state by inducing oxidative pathways that generate energy while inhibiting synthesis and growth pathways, thereby returning the cell to a state of energy homeostasis [26]. To achieve this regulation, AMPK targets a number of downstream pathways including those involved in lipid metabolism.

As a potent regulator of lipid metabolism, AMPK activity inhibits both sterol and fatty acid synthesis, while promoting fatty acid degradation [18]. AMPK directly phosphorylates acetyl-CoA carboxylase (ACC) and HMG-CoA Reductase (HMGCR), thereby inactivating these rate limiting enzymes in the metabolism of fatty acids and sterols respectively [36], [37]. In particular, ACC catalyzes the irreversible conversion of acetyl-CoA to malonyl-CoA, a key metabolite that plays multiple roles in fatty acid metabolism. First, malonyl-CoA is the substrate for fatty acid biogenesis, which drives *de novo* production of the fatty acid palmitate [38]. Second, malonyl-CoA is a co-substrate for chain lengthening of endogenously synthesized and dietary-derived essential fatty acids into higher polyunsaturated fatty acids [39]. Third, malonyl-CoA binding inhibits carnitine palmitoyltransferase I (CPT-1), an essential factor in the transport of fatty acids to the mitochondria for beta oxidation [38]. Thus malonyl-CoA production by ACC promotes fatty acid synthesis, while inhibiting fatty acid oxidation. Mammalian systems encode two non-redundant ACC isoforms, ACC1 and ACC2, which are both inactivated by AMPK-mediated phosphorylation. Studies suggest that malonyl-CoA produced by ACC2 is involved in fatty acid oxidation, while ACC1 contributes to fatty acid biogenesis [18]. Therefore, activation of AMPK through stress or low energy conditions induces fatty acid oxidation through ACC2, while inhibiting fatty acid synthesis through ACC1, with a net result of lipid breakdown.

We found that AMPK is potently antiviral against RVFV, and this restriction is dependent on the upstream activator LKB1. Furthermore, pharmacological activation of AMPK inhibited viral infection. AMPK was activated by RVFV infection, and in particular we observed striking changes in ACC activity dependent on AMPK, leading us to discover that AMPK is antiviral through its role in fatty acid metabolism. Cells lacking AMPK had increased global lipid levels, while pharmacological activation of AMPK led to decreased cellular lipids, consistent with AMPK control of lipid availability as a restriction point for viral replication. Importantly, we could bypass the antiviral effects of AMPK by feeding cells palmitate, the first fatty acid produced downstream of ACC. Since palmitate treatment restored RVFV infection, we demonstrate that AMPK specifically restricts infection through its role in inhibiting fatty acid biosynthesis. Since many viruses are dependent upon fatty acid biosynthesis for their replication, we tested whether AMPK restricted additional RNA viruses. We found that indeed, AMPK has antiviral activity against multiple arboviruses from disparate families including: the Flavivirus Kunjin virus, the Togavirus Sindbis virus, and the Rhabdovirus Vesicular stomatitis virus. Taken together, our data suggest that AMPK activation is broadly anti-viral, and may provide a novel antiviral therapeutic target.

## Results

### AMPK Restricts RVFV Infection

**Figure 1:**
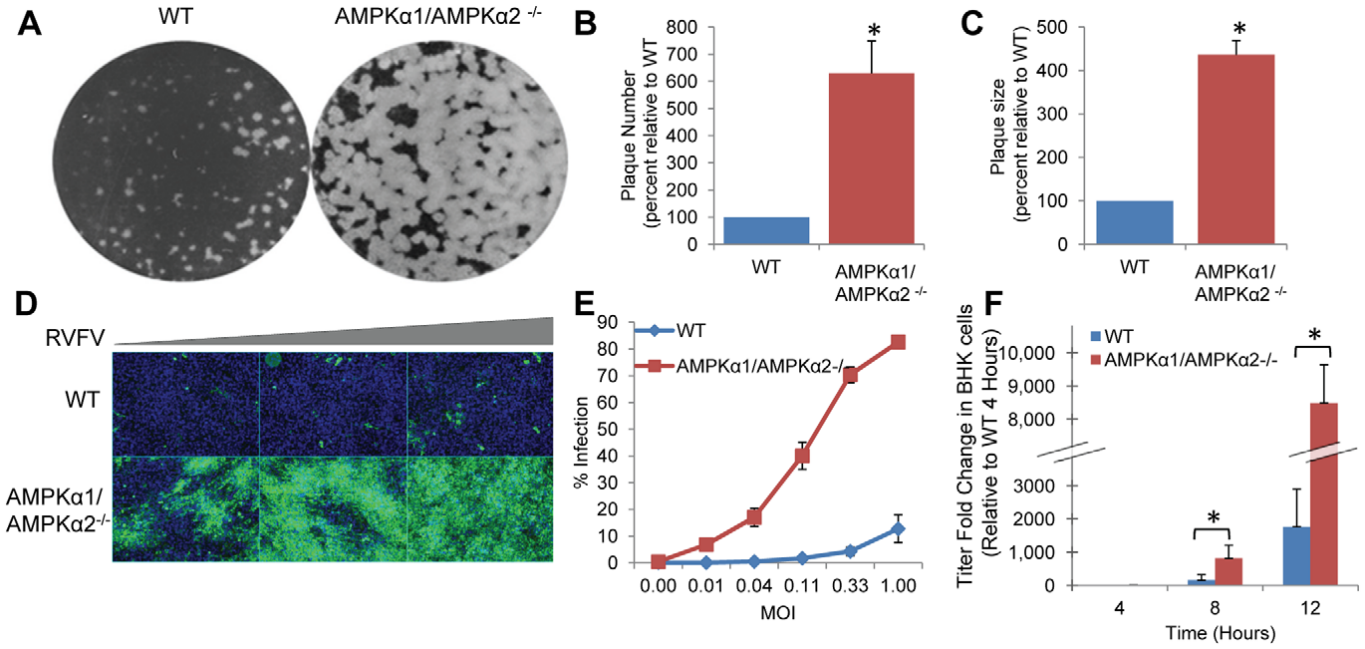
AMPK restricts RVFV infection. **A.** Plaque assays were performed on wild type (WT) and AMPKα1/AMPKα2^−/−^ MEFs. Representative data from triplicate experiments is shown. **B.** Quantification of plaques from **A.** presented as the normalized mean±SD relative to the number of wild type plaques from three experiments. **C.** The diameter of 30 representative plaques in each duplicate well from **A.** was used to calculate the average plaque area, displayed as the normalized mean+SD in triplicate experiments. **D.** WT or AMPKα1/AMPKα2^−/−^ MEFs were infected with serial dilutions of RVFV, incubated for 16 hours, and processed for immunofluorescence. (RVFV-N, green; nuclei, blue). **E.** Quantification of **D.** presented as percent of infected cells. A representative of three experiments is shown. **F.** One-step growth curve of RVFV in WT or AMPKα1/AMPKα2^−/−^ MEFs. RVFV grown in WT or AMPKα1/AMPKα2^−/−^ MEFs for 4, 8, or 12 hours was tittered on BHK cells and is presented as the normalized mean of triplicate experiments ±SD. * indicates p<0.05.

We previously reported that AMPK was required for efficient vaccinia infection through its role in macropinocytosis [40]. This led us to investigate the role of AMPK in other virus infections; we were particularly interested in RVFV as it is a virus that is medically important, but little is known about the mechanisms by which it establishes a productive infection. For our studies we used the lab adapted strain MP12 that has 11 amino acid differences from the wild type strain, since the wild type strain must be used in high containment facilities [41]. In order to test the role of AMPK in RVFV infection, we took advantage of mouse embryonic fibroblasts (MEF) that are genetically altered and null for both of the catalytic α subunits, AMPKα1 and AMPKα2 (AMPKα1/AMPKα2^−/−^) [42]–[44]. We challenged either the AMPKα1/AMPKα2^−/−^ MEFs or their sibling control wild type MEFs with RVFV and measured infection by plaque assay (Figure 1A). We found an increase in titer from 5×10^5^ pfu/ml to 3×10^6^ pfu/ml, indicating a 6-fold increase in the number of plaques formed in AMPKα1/AMPKα2^−/−^ MEFs compared to wild type (Figure 1B), concomitant with a 4-fold increase in average plaque area in AMPKα1/AMPKα2^−/−^ MEFs (Figure 1C). Moreover, RVFV infection was also increased in AMPKα1/AMPKα2^−/−^ MEFs as measured by an immunofluorescence assay that detects production of the RVFV N protein produced during viral replication (Figure 1D, quantified in Figure 1E), indicating that RVFV is able to infect and spread more efficiently in the absence of AMPK. Consistent with a role for AMPK both in early events during viral replication and in spread as measured by plaque assay Figure 1A), we observed an increase in viral infection at early time points before virus spread, as well as increased spread in cells lacking AMPK by monitoring the production of RVFV N protein over time by microscopy (Figure S1A–B).

This increased spread, indicated by the increase in plaque size (Figure 1C), as well as the immunofluorescence assay (Figure S1A–B), could result from increased production of infectious virus or increased infectivity of the virions produced in cells lacking AMPK. We measured the amount of infectious virus produced in wild type and AMPKα1/AMPKα2^−/−^ MEFs over time in a one-step growth curve. Medium from infected cells was collected at various times after infection, and virus was tittered on wild type BHK cells. Little virus (less than 1×10^4^ pfu/ml) was detected at 2–4 hpi, indicating that input virus was not detected in this assay (Figure 1F). Virus release began at 8 hpi, where we already observed an 8-fold increase in titer in the AMPK deficient MEFs (1.6×10^5^ pfu/ml versus 1.3×10^6^) (Figure 1F). This increase in titer was also observed at 12 hpi. Therefore, the increase in RVFV spread is likely due to increased virus production in AMPKα1/AMPKα2^−/−^ MEFs.

### AMPK Activation Restricts RVFV

**Figure 2:**
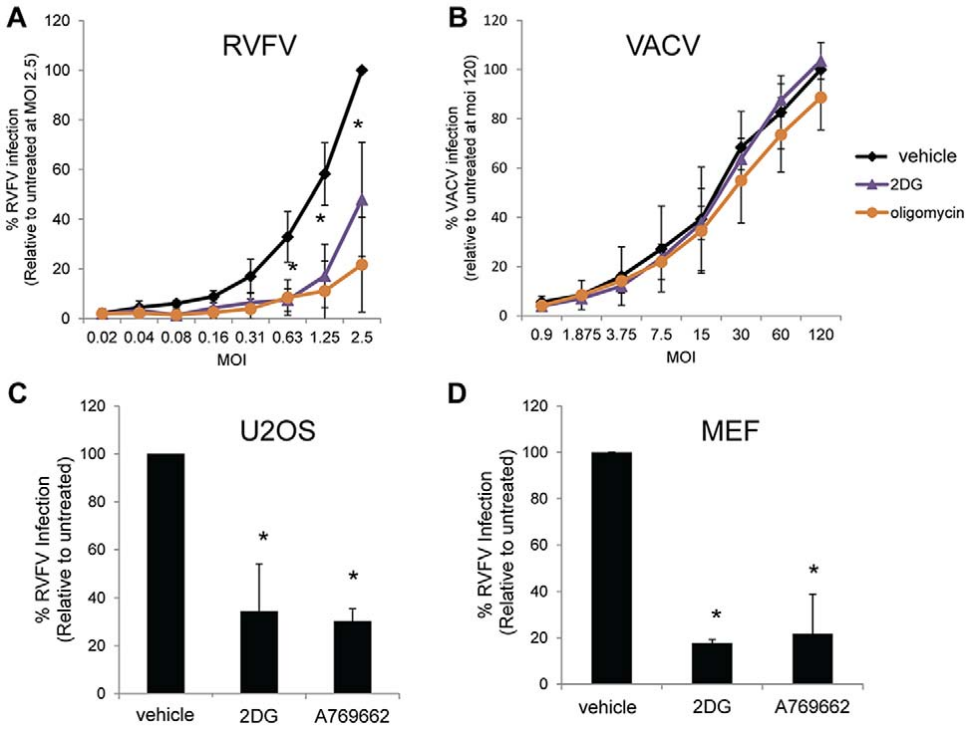
AMPK activation restricts RVFV. **A–B.** U2OS cells were pretreated with 10 mM 2DG, 10 µM oligomycin or PBS (untreated) for 1 hour and infected with serial dilutions of RVFV (**A**) for 10 hours or vaccinia virus (**B**) for 8 hours and processed for immunofluorescence. Data are displayed as the average percent infection relative to the highest concentration of virus in the untreated control ± SD from triplicate experiments. **C–D.** U2OS cells (**C**) or MEFs (**D**) were pretreated with 12 mM 2DG, 100 µM A769662, or PBS for 1 hour and infected with RVFV (MOI 1) for 10 hours. Infection was measured by immunofluorescence. Data are displayed as the normalized percent infection relative to the untreated control ±SD in triplicate experiments; * indicates p<0.05.

AMPK is activated through phosphorylation of a threonine residue on the catalytic alpha subunit [45]. Since AMPK deficiency increased RVFV infection, we hypothesized that AMPK activation would inhibit infection. Therefore, we tested whether RVFV was sensitive to pharmacological treatments that activate AMPK. First, we tested drugs that activate AMPK by reducing the levels of cellular energy using an independent cell line, the human osteosarcoma cell line (U2OS). We tested the glucose analog 2-deoxyglucose (2DG), and the ATP synthase inhibitor oligomycin, and found that both treatments significantly decreased infection by RVFV compared to vehicle controls (Figure 2A). In contrast, the AMPK inhibitor Compound C significantly, albeit modestly, increased RVFV infection (Figure S2A). Since 2DG and oligomycin activate AMPK indirectly by reducing cellular energy levels, and thus likely have other effects that may also contribute to viral infection, we tested whether these treatments affected vaccinia virus infection, which is not restricted by AMPK, but rather requires AMPK, independent of the energy sensing pathway for efficient viral infection [40]. Vaccinia virus infection was not affected by these treatments (Figure 2B), indicating that the compound-treated cells remain healthy enough to support viral infection, and the reduced infection levels were specific to RVFV. Moreover, we found that none of these drug treatments reduced cell number by greater that 20%, and therefore were not cytotoxic (Figure S2B).

Next, we took advantage of a recently developed thienopyridone compound A769662 that activates AMPK directly, independently of the energy status of the cell [46], [47]. This drug mimics both allosteric activation of AMPK and inhibition of dephosphorylation without affecting binding of AMP to the gamma subunit [48]. We found that RVFV infection of U2OS cells was significantly reduced in the presence of this compound (Figure 2C), and that both 2DG and A769662 inhibit RVFV in a dose-dependent manner (Figure S3A–B), indicating that AMPK activation restricts RVFV infection independently of the pleiotropic effects of reduced cellular energy levels. Moreover, we also found that the AMPK activating drugs 2DG and A769662 significantly inhibit RVFV infection in MEFs (Figure 2D). To determine if the effects of these drugs was specific for AMPK we treated AMPKα1/AMPKα2^−/−^ MEFs with the direct AMPK activator A769662. Treatment with this drug inhibited RVFV less than 2 fold in AMPKα1/AMPKα2^−/−^ MEFs and was not significant, whereas infection was inhibited greater than 5-fold in the wild type cells (Figure S4A) with no toxicity in either cell type (Figure S4B), indicating that the major action of this drug was through AMPK as previously published [46], [47]. Taken together, these studies suggest that AMPK activation has antiviral activity against RVFV in multiple cell types.

### LKB1 Restricts RVFV Infection

**Figure 3:**
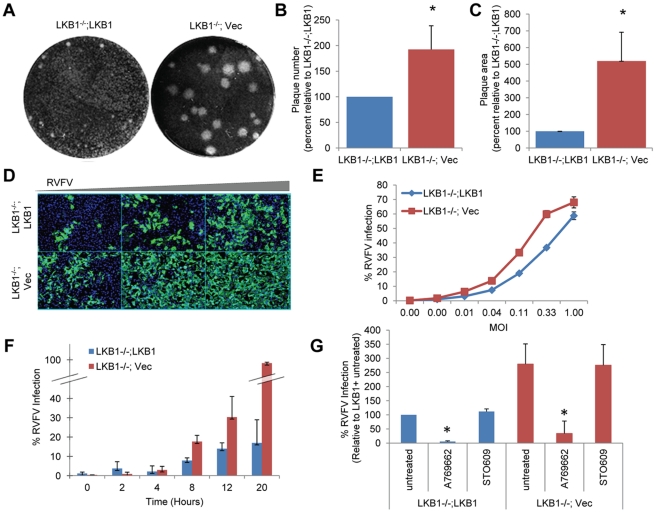
LKB1 restricts RVFV infection. **A.** RVFV was plaqued on LKB1−/−;LKB1 and LKB1−/−;Vec MEFs. Representative data from triplicate experiments is shown. **B.** Quantification of plaques from **A.** presented as the normalized mean±SD of wild type plaques from three experiments. **C.** The diameter of 30 representative plaques each of three experiments was used to calculate the average plaque area, which is displayed as the normalized mean±SD in triplicate experiments. **D.** LKB1−/−;LKB1 or LKB1−/−;Vec MEFs were infected with serial dilutions of RVFV, incubated for 16 hours, and processed for immunofluorescence. (RVFV-N green; nuclei blue). A representative of triplicate experiments is shown. **E.** Quantification of **D.** presented as RVFV percent infection in LKB1−/−;LKB1 and LKB1−/−;Vec MEFs. A representative of triplicate experiments is shown. **F.** Time course of RVFV infection in LKB1−/−;LKB1 and LKB1−/−;Vec MEFs. Cells were infected with RVFV (MOI 1), and fixed at indicated hours post infection. A representative of triplicate experiments is shown. **G.** LKB1−/−;LKB1 or LKB1−/−;Vec MEFs were pretreated with 100 µM A769662 or 10 µg/ml STO609 for 1 hour prior to infection with RVFV (MOI 1) for 10 hours and processed for immunofluorescence. Data are displayed as the average percent infection relative to the LKB1−/−;LKB1 untreated control ± SD from triplicate experiments. * indicates p<0.05.

Since pharmacological activation of AMPK restricted RVFV infection, we were interested in investigating which pathway upstream of AMPK was responsible for this restriction. The classic activator of AMPK is the tumor suppressor LKB1, which phosphorylates AMPK in response to a variety of stimuli that cause a reduction in cellular energy levels, such as glucose starvation or hypoxia [26]. In order to determine if LKB1 signaling was important for AMPK-mediated RVFV restriction, we tested whether LKB1 also restricted RVFV. We challenged MEFs that are null for LKB1 and complemented with either vector alone (LKB1^−/−^; Vec), or an LKB1 cDNA (LKB1^−/−^; LKB1) [40] and found increased RVFV infection in MEFs lacking LKB1 by plaque assay (Figure 3A). Quantification revealed a 2-fold increase in the number of plaques (increase in average virus titer from 7.8×10^5^ to 1.5×10^6^ pfu/ml in LKB1 null MEFs) (Figure 3B) and a 5-fold increase in plaque area in LKB1^−/−^; Vec MEFs compared to MEFs complemented with LKB1 (Figure 3C). Moreover, we observed increased infection in the LKB1^−/−^; Vec MEFs compared to those complemented with LKB1 by immunofluorescence (Figure 3D, quantified in 3E). Finally, we measured RVFV infection over time in cells lacking LKB1 and found increased infection in the absence of LKB1 at early and late times after infection, indicating increased initial infection as well as spread (Figure 3F). Since AMPK activation downstream of LKB1 is dependent on a decrease in cellular energy, we measured cellular ATP levels during RVFV infection using a luciferase assay. While 2DG significantly reduced cellular ATP levels, neither A769662 nor RVFV had any impact on ATP levels as measured by this assay (Figure S5). While infection with RVFV did not induce global changes in cellular ATP, this does not rule out localized changes in cellular energy that could influence AMPK.

In addition to LKB1 other upstream activators of AMPK have been identified. Notably, calcium-calmodulin kinase kinase (CaMKK) has been shown to activate AMPK in response to an increase in intercellular calcium [33], [34], [49]. Since LKB1 did not restrict RVFV as strongly as AMPK did (Figure 3), we investigated if other upstream activators, such as CaMKK could also contribute to RVFV restriction. To this end, we treated U2OS cells with the CaMKK inhibitor STO609 prior to infection, and found no increase in RVFV infection in response to this drug, although at very high concentrations there was a decrease in infection (Figure S3C). This decrease was likely due to additional kinases that are inhibited at these concentrations [50]. This finding is consistent with previous reports that changes in intercellular calcium levels are not induced by RVFV infection [51]. We next investigated if LKB1 and CaMKK function redundantly to restrict RVFV infection. We tested whether simultaneously inhibiting both LKB1 and CaMKK would lead to a greater increase in RVFV infection than LKB1 deficiency alone. To this end, prior to infection, we treated LKB1 null MEFs or those complemented with LKB1 with STO609 and monitored RVFV infection. Consistent with our previous findings, we observed a 3-fold increase in the percentage of infected cells in LKB1 null cells compared to those complemented with LKB1; however pretreatment with STO609 had no effect on infection level in either cell type (Figure 3G). In contrast, and as expected, we found that pretreatment with the AMPK activating compound A769662 significantly inhibited RVFV in both LKB1 null and complemented MEFs (Figure 3G). Taken together, these data suggest that LKB1 is the major upstream activator responsible for AMPK-mediated restriction of RVFV.

### AMPK Restricts RVFV RNA Replication

**Figure 4:**
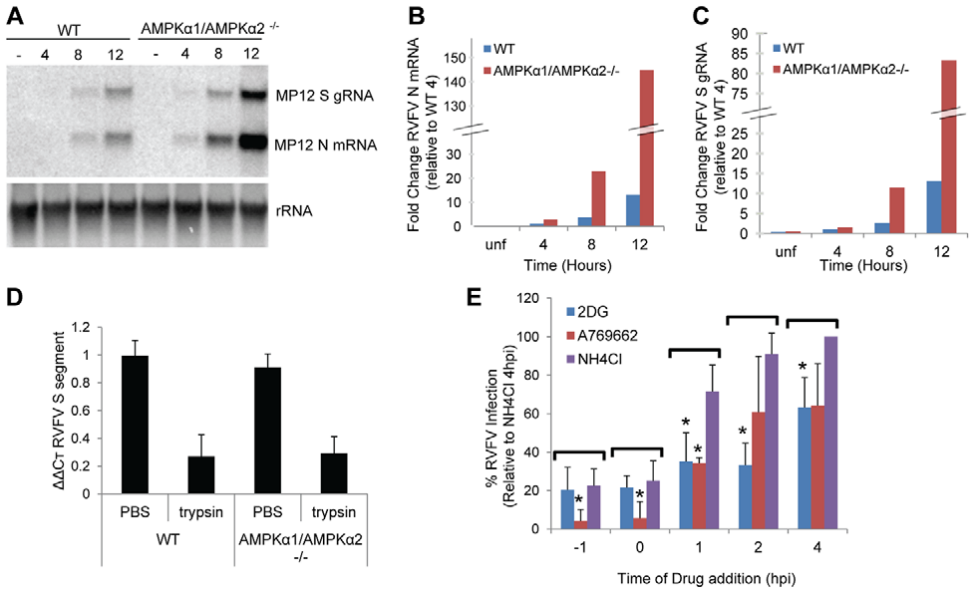
AMPK restricts RVFV RNA replication. **A.** Northern blot of genomic S segment and N mRNA from RVFV (MOI 1) grown in WT or AMPKα1/AMPKα2^−/−^ MEFs for 4, 8, or 12 hours. A representative of triplicate experiments is shown. **B–C.** Quantification of RVFV mRNA (**B**) or genomic RNA (**C**) in WT or AMPKα1/AMPKα2^−/−^ MEFs displayed as the normalized fold change from WT 4 hours. A representative of triplicate experiments is shown. **D.** RVFV binding assay. RVFV (MOI 10) was bound to WT or AMPKα1/AMPKα2^−/−^ MEFs at 4°C for 1 hour, then washed, and treated with PBS or trypsin to remove bound virus. qRTPCR was performed on isolated RNA to detect RVFV S genome. Data are displayed as the average ΔΔCT of triplicate experiments normalized to GAPDH control. * indicates p<0.05. **E.** 2DG (12 mM), A769662 (100 uM) or Ammonium Chloride (NH_4_Cl, 12 mM) was added either 1 hour prior to infection with RVFV (MOI 1), with infection, or 1, 2, or 4 hours post infection. After 10 hours of infection cells were fixed and processed for immunofluorescence. Data are displayed as the average percent infection relative to the post entry level of infection (NH_4_Cl added at 4 hpi) ± SD from triplicate experiments. * indicates p<0.05.

To dissect the mechanism by which AMPK restricts RVFV infection, we first determined which early step in the viral replication cycle is restricted by AMPK. We observed decreased protein production, as measured by immunofluorescence (Figure 1D–E and Figure S1) in addition to decreased production of infectious progeny virus (Figure 1F) in the presence of AMPK. This suggests that AMPK may inhibit a step in the viral replication cycle at, or prior to, protein production. To determine if viral RNA replication was affected by AMPK, we monitored both viral genomic RNA replication and viral mRNA production in the presence or absence of AMPK. We found an increase in both viral mRNA (N) and genomic RNA (S segment) in AMPK deficient MEFs both early in infection and upon virus spread (Figure 4A–C). At 4 hpi, a time point prior to RVFV release, we observed a 3-fold increase in viral mRNA production in AMPK deficient MEFs compared to wild type, which continued to increase over time (Figure 4A–B). Likewise, genomic RNA production was increased prior to virus release and spread (Figure 4A and C). These data suggest that the increased N protein production observed by immunofluorescence at early time points (Figure S1A) may be due to increased N mRNA production.

Next, we investigated whether entry, a step upstream of RNA replication, was inhibited by AMPK. First, we tested whether RVFV binding was more efficient in the absence of AMPK. To this end, MEFs were pre-bound with RVFV for an hour at 4°C, unbound virus was removed and RVFV binding was measured by quantitative RT-PCR to detect genomic RVFV S segment within virions. We observed no difference in virus binding in wild type or AMPK deficient cells (Figure 4D). Moreover, the majority of virus was removed by trypsin treatment in both wild type and AMPK deficient MEFs, indicating these virions had bound to the cell surface, but not entered (Figure 4D).

Since AMPK did not impede virus binding, we next performed a time of addition assay to test whether AMPK-activating drugs restricted entry. Since Bunyaviruses such as RVFV enter cells through a pH-dependent route of endocytosis [51]–[53], we used the lysosomotropic agent ammonium chloride, which raises the pH of lysosomal compartments, to define the timing of virus entry. Ammonium chloride inhibited infection strongly (to 20% of the 4 hpi addition) when added 1 hour prior to infection or with infection (t = 0); however, by 1 hpi, more than 70% of infection had returned, indicating that the majority of RVFV had entered by this time point (Figure 4E). Thus we compared each treatment to the post entry level of RVFV infection (ammonium chloride added at 4 hpi). AMPK activating drugs 2DG, and A769662 significantly inhibited infection when added at post entry stages (Figure 4E); however, since one of the AMPK activating drugs, A769662, had a significantly greater impact on RVFV when added prior to or with infection, we cannot rule out that AMPK also inhibits RVFV entry. Taken together these data suggest that AMPK restricts RVFV during initial stages of replication post entry, likely at the step of RNA replication. This reduction in viral RNA and protein production likely leads to a reduction in release of infectious virus and spread observed at later stages of infection.

### The Antiviral Effects of AMPK Are Independent of Type I Interferon

The classical cell-mediated response to viral infection is the type I interferon system [54], [55]. Therefore, we investigated whether AMPK impacts the expression of interferon beta (IFNβ) or its downstream effector 2′-5′-oligoadenylate synthetase 1 (OAS1) by qRT-PCR. We found that RVFV infection induced both IFNβ and OAS1 in both wild type and AMPK deficient cells although the basal levels and induction of these genes were higher in cells lacking AMPK (Figure S6A–B). This result was opposite to what would have been predicted, if IFNβ induction was responsible for the antiviral phenotype. In addition, we tested whether IFNβ treatment induced AMPK or ACC phosphorylation and found that it did not (Figure S6C, quantified in D). Altogether, these data indicate that AMPK has antiviral activity independent of the classical type I IFN response.

### Acetyl-CoA Carboxylase Activity Is Tightly Regulated by AMPK during RVFV Infection

**Figure 5:**
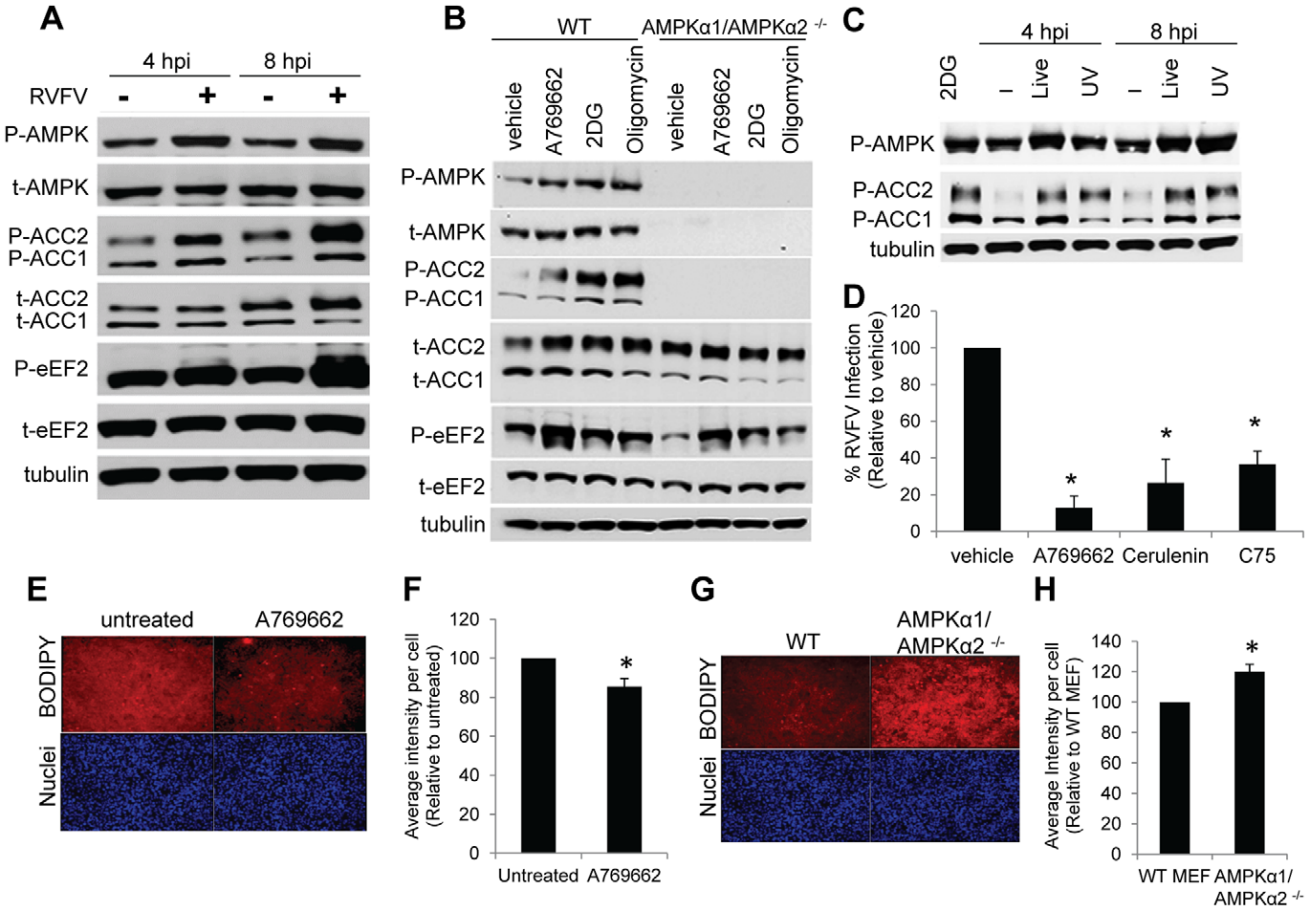
Acetyl-CoA Carboxylase Activity is Tightly Regulated by AMPK during RVFV Infection. **A.** Phosphorylation of AMPK and downstream effectors upon RVFV infection. WT MEFs were infected with RVFV (MOI 1) for 4 or 8 hours. Lysates were collected and assayed by immunoblot for phospho-AMPK, phospho-ACC, and phospho-eEF2. Total protein was assayed for each and Tubulin was measured as a loading control. Representative blot of triplicate experiments is shown. **B.** Phosphorylation of AMPK and downstream effectors in WT and AMPKα1/AMPKα2^−/−^ MEFs. Cells were treated with AMPK activators 2DG (12 mM), oligomycin (OM, 10 µM), and A769662 (100 µM) for 4 hours. Lysates were collected and assayed by immunoblot as above. Representative blot of triplicate experiments shown. **C.** Phosphorylation of AMPK and ACC upon treatment with UV-inactivated RVFV. WT MEFs were infected with live or UV-inactivated RVFV (MOI 1) for 4 or 8 hours. Lysates were collected and assayed by immunoblot as above. Representative blot of triplicate experiments is shown. **D.** Blocking fatty acid synthesis inhibits RVFV infection. MEFs were treated with the fatty acid synthase inhibitors Cerulenin (45 pM) and C75 (12.5 µM) or the AMPK activator A769662 (100 µM), infected with RVFV (MOI 1), and processed for immunofluorescence. Data are displayed as the normalized average percent infection relative to the untreated control ± SD in triplicate experiments. * indicates p<0.05. **E.** WT MEFs were treated with 100 µM A769662 for 10 hours and stained for cellular lipids with BODIPY lipophilic fluorescent dye. (BODIPY, red; nuclei, blue). Representative images from triplicate experiments are shown. **F.** Quantification of **E.** presented as integrated BODIPY intensity per cell relative to untreated control ± SD in triplicate experiments. * indicates p<0.05. **G.** WT and AMPKα1/AMPKα2^−/−^ MEFs were grown overnight and stained for cellular lipids with BODIPY lipophilic fluorescent dye. (BODIPY, red; nuclei, blue). Representative images from triplicate experiments are shown. **H.** Quantification of **G.** presented as integrated BODIPY intensity per cell relative to WT ± SD in triplicate experiments. * indicates p<0.05.

Since AMPK activation has antiviral activity against RVFV, we examined whether AMPK is activated by RVFV infection. To this end, we measured AMPK phosphorylation at Thr172 by immunoblot. AMPK phosphorylation was increased at 4 and 8 hours after infection compared to uninfected controls (Figure 5A, quantified in Figure S7A), indicating that RVFV infection induced AMPK activation. Furthermore, we found that UV-irradiated virus, incapable of replication (Figure S8), also induced AMPK phosphorylation at 4 and 8 hours after treatment (Figure 5C), suggesting that activation was triggered by incoming virus particles and viral replication was not required. Finally, we confirmed that LKB1 was required for RVFV-dependent activation of AMPK (Figure S9).

AMPK regulates several downstream pathways that could be important for viral infection, in particular protein translation and lipid synthesis [56]. Thus, we examined the activation status of two classical downstream effectors of AMPK involved in translation and lipid biosynthesis which are inactivated by AMPK-mediated phosphorylation [26]. Elongation Factor 2 (eEF2) is an important regulator of translation elongation, and Acetyl-CoA Carboxylase (ACC) consists of two enzymes involved in fatty acid metabolism (ACC1 and ACC2) [38], [57]. Both eEF2 and ACC had increased levels of phosphorylation at 4 and 8 hours after infection with RVFV compared to uninfected controls, consistent with the activation status of AMPK (Figure 5A, quantified in Figure S7B–D). Little difference in total protein levels of AMPK, ACC or eEF2 was observed during infection. Taken together, these data suggest that RVFV infection leads to increased AMPK signaling.

To explore the mechanism by which AMPK restricts RVFV replication, we examined the impact of AMPK on translation and lipid biogenesis, both of which contribute to important steps in virus infection. In particular, AMPK inhibits translation initiation by inactivating mTORC1, and translation elongation by inactivating eEF2 [58]–[60]. Inactivation of mTORC1 by AMPK leads to decreased translation initiation as well as increased autophagy, both of which could have anti-viral effects [58]. Since AMPK activation inhibits mTORC1 activity, we hypothesized that mTORC1, and thus protein synthesis, would be overactive in AMPK deficient cells, perhaps allowing for increased viral protein production and replication. We tested the requirement for mTORC1 signaling in RVFV infection using the mTORC1 inhibitor Rapamycin, and found no significant difference in RVFV infection in cells treated with Rapamycin compared to vehicle controls in either wild type or AMPKα1/AMPKα2^−/−^ MEFs (Figure S10A). This finding suggests that the antiviral activity of AMPK is independent of mTORC1 signaling. Furthermore, since AMPK activation can increase autophagy, which has been shown to have antiviral effects in some models [61], we tested whether inhibition of autophagy impacted RVFV infection by plaque assay, and found no significant difference in MEFs expressing a ATG5 hairpin, which knocks down ATG5, compared to control MEFs (Figure S10B–C).

Next we investigated whether reduced translation elongation through eEF2 inactivation could be responsible for AMPK's antiviral activity against RVFV (Figure 5A). Since eEF2 is regulated by multiple upstream pathways in addition to AMPK, we first determined the sensitivity of eEF2 to AMPK regulation. In wild type MEFs, treatment with the AMPK activating drugs 2DG, oligomycin, or A769662 led to increased phosphorylation of AMPK, as well as downstream effectors eEF2 and ACC (Figure 5B, quantified in Figure S7E–H), as expected. As a control, we found that AMPK deficient MEFs did not express phosphorylated AMPK or total AMPK under any treatment condition. Interestingly, we observed an increase in phosphorylated eEF2 in response to all three drugs in AMPKα1/AMPKα2^−/−^ MEFs (Figure 5B, quantified in Figure S7H). In contrast, while we observed an increase in ACC phosphorylation in response to drug treatments in wild type MEFs, phosphorylated ACC was undetectable in AMPK deficient MEFs both basally and in response to treatment with AMPK activating compounds (Figure 5B). These phenotypes were not due to changes in total protein levels as they remained unchanged under all treatment conditions; although the AMPK deficient MEFs had a slightly lower basal level of ACC (Figure 5B). These findings suggest signaling pathways other than AMPK are important in regulating eEF2 phosphorylation, while ACC phosphorylation is exquisitely regulated by AMPK.

Given this observation, we pursued ACC as a potential regulator of antiviral defense. ACC is the first rate-limiting enzyme and master regulator of fatty acid metabolism, both by inhibiting fatty acid biosynthesis and activating fatty acid catabolism through beta-oxidation [18], [38]. Fatty acid biosynthesis is an important component of viral infection since numerous RNA viruses, including Bunyaviruses, proliferate cellular membrane structures for proper formation of the viral replication complex, in addition to using cellular membranes for their lipid coats [6], [14], [15], [17]. In order to assess the importance of fatty acid synthesis in RVFV infection, we tested the ability of RVFV to replicate within cells pretreated with the fatty acid synthase inhibitors. Fatty acid synthase is the next enzyme in fatty acid metabolism, using the product of ACC to generate palmitate, and thus is required for all fatty acid biosynthesis [62]. We observed a 5-fold decrease in RVFV infection in the presence of fatty acid synthase inhibitors cerulenin and C75 by immunofluorescence, similar to the decrease observed in cells pretreated with the AMPK activator A769662 (Figure 5D), indicating that *de novo* fatty acid synthesis is an important step early in RVFV infection.

ACC is the enzyme that converts acetyl-CoA into malonyl-CoA, a precursor in the synthesis of palmitate, the first product of *de novo* fatty acid biosynthesis. Since AMPK activation inhibits *de nov*o fatty acid synthesis by inactivating ACC, we tested whether altered levels of AMPK activation or expression affected cellular lipid levels. To this end, we stained MEFs with the lipophilic BODIPY fluorescent dye. We found that treatment with the AMPK activator A769662 led to a decrease in BODIPY staining compared to untreated MEFs (Figure 5E, quantified in F), consistent with decreased fatty acid synthesis during AMPK activation. In contrast, MEFs lacking AMPK had increased BODIPY staining compared to wild type cells (Figure 5G, quantified in H). These findings are consistent with previous reports that AMPK activating drugs, such as A769662 increase levels of beta-oxidation while decreasing fatty acid synthesis [46], [63], [64], and suggest that the absence of AMPK leads to overproduction of cellular lipids, while AMPK activation globally reduces cellular lipid levels.

### Palmitate Rescues AMPK-Mediated Restriction of RVFV

**Figure 6:**
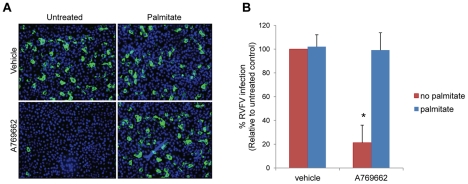
Addition of palmitate restores RVFV infection in the presence of A769662. **A.** U2OS cells were pretreated with 100 µM palmitate overnight and 100 µM A769662 or PBS was added 1 hour prior to infection with RVFV (MOI 1). Cells were incubated for 10 hours, and processed for immunofluorescence. (RVFV-N, green; nuclei, blue) **B.** Quantification of **A.** Data are displayed as the normalized percent infection relative to the untreated control at MOI 1.25±SD in triplicate experiments; * indicates p<0.05 compared to untreated vehicle control.

If AMPK activation restricts RVFV infection by reducing levels of fatty acid synthesis, exogenous addition of fatty acids should restore infection. Therefore, we tested whether we could bypass the requirement for AMPK-regulated fatty acid synthesis by pretreating cells with palmitate, the first product of fatty acid biosynthesis. We treated U2OS cells with palmitate overnight, and then added A769662 1 hour prior to infection with RVFV to activate AMPK. After 10 hours of infection, cells were fixed and stained for RVFV to measure percent infection in an immunofluorescence assay that monitors the initial round of infection. In cells treated with the AMPK activator A769662 alone, we found a 5-fold decrease in RVFV infection, consistent with our previous findings (Figure 6A, quantified in 6B). However, addition of palmitate prior to treatment with A769662 was able to restore infection to levels seen in untreated cells (Figure 6A, quantified in 6B). We observed a 5-fold increase in RVFV infection in cells treated with A769662 and palmitate compared to those treated with A769962 alone (Figure 6B), while addition of palmitate alone had little effect on infection (Figure 6A–B). Since chronic exposure to high concentrations of palmitate has previously been reported to inhibit AMPK activation, we confirmed by immunoblot that AMPK phosphorylation was not inhibited by the concentrations of palmitate used in our assay (Figure S11). Together, these data suggest that AMPK restricts RVFV infection primarily through inhibiting fatty acid biosynthesis.

### AMPK Restricts Multiple Arboviruses

**Figure 7:**
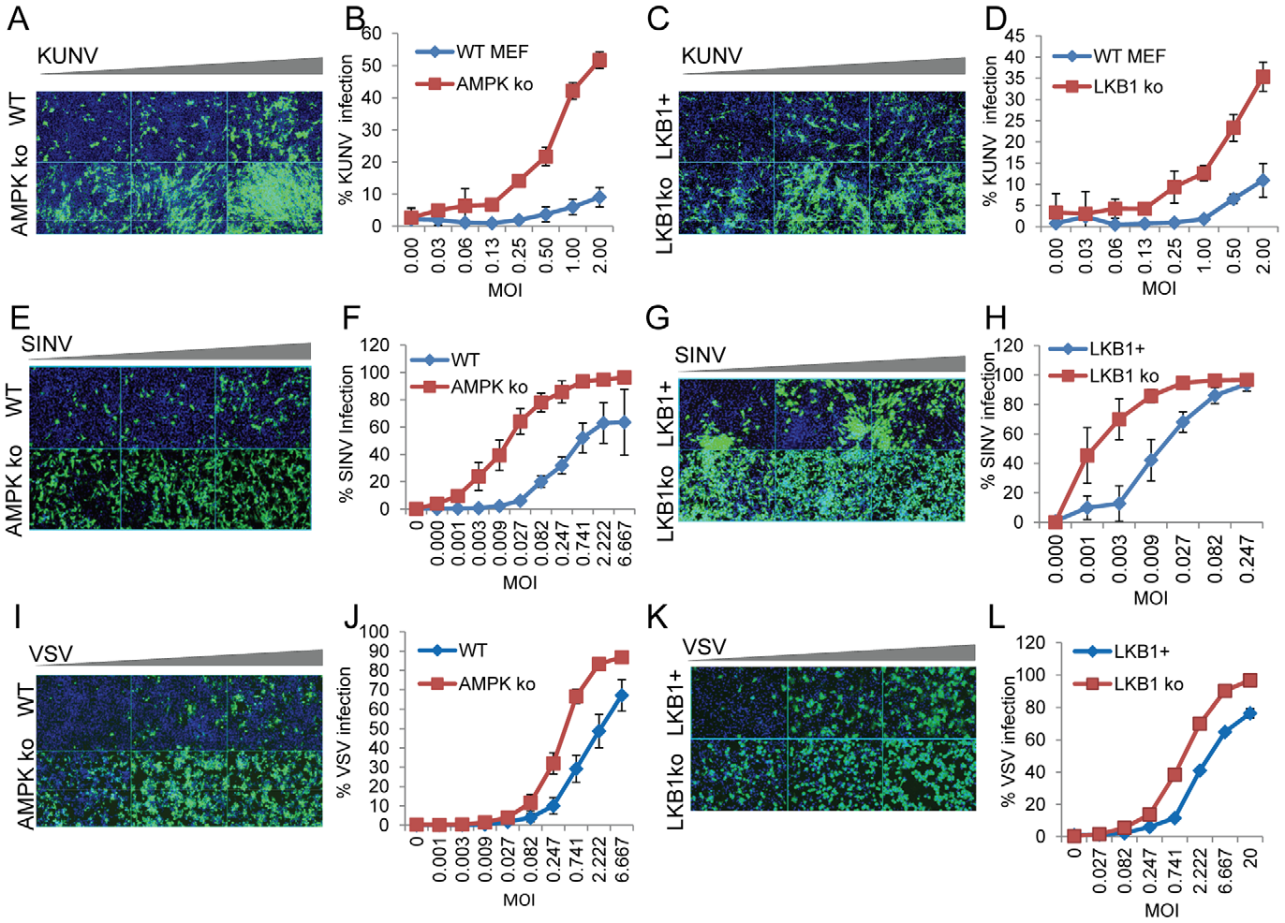
Additional arboviruses are restricted by AMPK. WT or AMPKα1/AMPKα2^−/−^ MEFs were infected with serial dilutions of KUNV (**A**), SINV (**E**), or VSV (**I**) and processed for immunofluorescence. (Virus, green; nuclei, blue). Quantifications of the percent infection for KUNV (**B**), SINV (**F**) and VSV (**J**) are shown as representatives of triplicate experiments. LKB1^−/−^;LKB1 and LKB1^−/−^;Vec MEFs were infected with serial dilutions of KUNV (**C**), SINV (**G**), and VSV (**K**) and processed for immunofluorescence. (Virus, green; nuclei, blue). Quantifications of the percent infection are shown for KUNV (**D**), SINV (**H**) and VSV (**L**) are shown as representatives of triplicate experiments.

A dependence on lipid biosynthesis and virally induced membrane modifications is not unique to Bunyaviruses; many RNA viruses require extensive membrane modifications and proliferations to support their replication complex [6], [65]. Therefore, we tested whether AMPK restricts additional arboviruses. To this end we tested the ability of the Flavivirus Kunjin virus (KUNV), the Togavirus Sindbis virus (SINV), and the Rhabdovirus Vesicular stomatitis virus (VSV) to grow in wild type and AMPKα1/AMPKα2^−/−^ MEFs by immunofluorescence. KUNV (Figure 7A–B), SINV (Figure 7E–F) and VSV (Figure 7I–J) had increased infections in AMPKα1/AMPKα2^−/−^ MEFs compared to wild type MEFs. Moreover, KUNV (Figure 7C–D), SINV (Figure 7G–H), and VSV (Figure 7K–L) infections were also increased in LKB1^−/−^; Vec compared to MEFs expressing LKB1, indicating that both AMPK and its canonical upstream activator LKB1 restrict additional arboviruses. Moreover, we have found that KUNV is also sensitive to the AMPK activator A769662, and can be partially rescued by palmitate addition (Figure S12A–B), although palmitate treatment itself decreased KUNV infection (Figure S12C). These data suggest that AMPK may restrict multiple RNA viruses by limiting fatty acids. Taken together our data suggest that AMPK is broadly anti-viral across disparate virus families, and may represent a novel cellular target for anti-viral therapeutics.

## Discussion

Arboviruses represent a group of emerging pathogens of both medical and agricultural importance for which there are few therapies. RVFV is a particularly important member of this group that causes disease both in humans and livestock, and is considered a Category A pathogen due to its high pathogenesis and potential for geographical spread. Here, we identified AMPK as a novel antiviral factor that restricts RVFV infection independent of the type I IFN system. This restriction is dependent on the canonical upstream activator LKB1. Furthermore, we found that AMPK is activated by RVFV infection, and this activation restricts infection at the level of RNA replication likely by reducing fatty acid biosynthesis, an essential process in RVFV infection. We extended these studies by demonstrating that additional arboviruses, known to require lipid biosynthesis, were also restricted by this pathway. Since treatment with drugs that activate AMPK restricted infection, this could represent a novel therapeutic strategy toward the control of many RNA viruses.

AMPK is a central regulator of cellular energy that regulates a number of cellular pathways that could influence viral replication, including protein and lipid biosynthesis [56]. AMPK activation inhibits protein translation through two major downstream pathways. First, AMPK activation inhibits translation initiation by inhibiting mTORC1 activity. Second, AMPK inhibits translation elongation through inactivation of eEF2. We explored these two targets as potentially regulating RVFV infection. However, we found RVFV was insensitive to treatment with the mTORC1 inhibitor, Rapamycin, regardless of AMPK status. Furthermore, eEF2 phosphorylation induced by drugs that alter the energy status of the cell was not affected in the absence of AMPK, indicating additional upstream regulators are contributing to eEF2 activity. Therefore, we explored lipid biosynthesis as a potential target for AMPK-dependent anti-viral activity.

AMPK controls fatty acid metabolism through ACC, and may be the only physiologically relevant kinase that controls ACC activity [18]. This is consistent with our findings that ACC phosphorylation was exquisitely dependent on AMPK, in contrast to eEF2, which was phosphorylated during energy depletion even in the absence of AMPK. ACC is the enzyme responsible for the conversion of acetyl-CoA to malonyl-CoA [38]. Malonyl-CoA production impacts lipid metabolism in at least three ways. Malonyl-CoA is a substrate driving *de novo* palmitate production, and is also important in converting simple essential fatty acids into more complex polyunsaturated fatty acids that can be used to build triglycerides and other cellular lipids [39]. Finally, malonyl-CoA inhibits transport of fatty acids to the mitochondria, thus inhibiting fatty acid oxidation [38]. In addition to its role in fatty acid metabolism, AMPK is also an important regulator of HMG-CoA reductase (HMGCR), the rate limiting enzyme in the synthesis of isoprenoids and sterols, including cholesterol. Cholesterol is known to contribute to infection of multiple viruses, and therefore could also be targeted in AMPK-mediated virus restriction.

Since we found that fatty acid biosynthesis was required for RVFV infection, and changes to AMPK expression and activation status led to global changes in cellular lipid levels, we hypothesized that inhibiting fatty acid synthesis downstream of ACC was responsible for AMPK-mediated restriction of RVFV. This was supported by our finding that we could bypass the requirement for malonyl-CoA production by introducing exogenous palmitate. Since the addition of palmitate rescued RVFV overcoming the restriction mediated by AMPK activation (Figure 6), the ability of AMPK to inhibit fatty acid biosynthesis is likely the most important determinant of AMPK-mediated RVFV restriction. Palmitate is a substrate for the biosynthesis of a number of lipid moieties that could contribute to RVFV infection. Palmitate undergoes chain elongation and additional modifications in the ER to produce saturated fatty acids as well as triglycerides, phospholipids, and cholesterol esters [66], [67]. It is also a substrate for sphingolipid biosynthesis in the Golgi. Sphingolipids become incorporated into cellular membranes and participate in signaling events that could contribute to RVFV infection. Finally, palmitate addition is a form of post-translational modification of some proteins [68].

There are several stages during the course of RVFV infection where cellular lipids are utilized. Many RNA viruses induce the formation of novel membranous structures derived from various organelles within the cell to support the viral replication complex [6]. Notably, formation of these structures is often dependent on *de novo* fatty acid synthesis [69]–[72]. While RVFV-induced membrane alterations have not been well characterized, a related Bunyavirus, Bunyamwera virus, was reported to induce Golgi-derived tubular structures with globular heads in association with the viral replication complex, suggesting that other Bunyaviruses could likewise induce membrane changes [14], [15]. In addition to RNA replication, enveloped viruses bud from cellular membranes, thereby incorporating those lipids into the viral particle [17]. RVFV assembly occurs on Golgi membranes, with virus particles ultimately budding into the Golgi for transport and release at the plasma membrane [73]. Cellular lipids derived from *de novo* palmitate production downstream of ACC could contribute to each of these steps, although our findings that viral RNA synthesis is inhibited by AMPK suggests that RNA replication is a key target.

In addition to RVFV, we found that three additional viruses including the Togavirus SINV, the Flavivirus KUNV, and the Rhabdovirus VSV are restricted by AMPK and LKB1 (Figure 7). Importantly, this group includes members of the three major families of arboviruses that contribute to human disease. Members of the Togavirus family including Semliki Forest virus and Rubella virus have been described to induce characteristic modified endosomal and lysosomal structures termed cytopathic vacuoles that support the viral replication complex [10], [11], [74], [75]. Furthermore, a number of Flaviviruses have been shown to have important lipid dependencies. KUNV, a strain of West Nile virus, has been described as forming two distinct membrane structures that include double membrane spherical vesicles that are the sites of viral replication, as well as arrays of convoluted membranes that are the sites of viral polyprotein processing [76]–[81]. Moreover, both fatty acid synthesis and oxidation have been shown to be essential for another Flavivirus, Dengue virus (DENV). Infection is characterized by virally-induced increases in cellular fatty acid synthesis and a redistribution of the enzyme fatty acid synthase to sites of DENV replication [70]. Free fatty acids are also derived through autophagosomal processing of triglycerides, and exogenous addition of the fatty acid oleate was able to rescue DENV infection when autophagy is inhibited [82]. Furthermore, induction of ER-derived lipid droplet formation is necessary for DENV particle formation [83]. Therefore DENV and perhaps many other viruses require complex and unique interactions with cellular lipid metabolism through both synthesis and degradation pathways. In addition, Hepatitis C Virus (HCV), a distantly related Flavivirus, induces formation of a membranous web derived from intracellular vesicles, whose formation requires fatty acid synthesis for replication [8], [9]. Interestingly, AMPK has been implicated to play a role in HCV infections. AMPK-activating drugs inhibited the replication of HCV replicons concomitant with a decrease in cellular lipid levels, while knock down of the upstream activator LKB1 led to increased replication, [84], consistent with our findings with RVFV, KUNV, SINV, and VSV. Importantly, KUNV could be partially rescued from AMPK-mediated restriction by the addition of the fatty acid palmitate. Thus, AMPK may restrict multiple families of viruses through this mechanism. Since all positive strand RNA viruses are thought to induce membrane modifications for viral RNA replication, and include a large number of medically significant groups (e.g., Picornaviruses, and Coronaviruses) [16], [80], [85], [86], it will be important to determine the full scope of viruses restricted by AMPK as well as the mechanism of restriction.

Since many disparate viruses are restricted by AMPK, it is interesting to speculate how AMPK could be activated in response to these viral infections. We have found that both live virus and UV-inactivated replication incompetent RVFV is capable of activating AMPK via LKB1. This suggests that the energy sensing pathway is responsible for this activation yet we were unable to detect global changes in cellular energy levels during the period in infection when AMPK becomes phosphorylated. Thus, we hypothesize that RVFV infection induces a localized drop in cellular energy to activate AMPK. Since this is independent of viral replication and can restrict a large panel of disparate viruses that have the commonality of entering cells via endocytic routes and fusing within these compartments, we postulate that a local energy drop may occur during these steps. Since endocytosis is a highly energetic process usurped by many viruses, it is possible that increased levels could themselves could provide the trigger for this rapidly inducible antiviral response. We have previously reported that receptor-mediated endocytosis, employed by many viruses including KUNV, SINV and VSV for entry is intact in AMPK deficient cells [40]. Therefore at least some routes of endocytic entry used by viruses are unaffected by AMPK, and may provide a trigger for activation rather than a point of restriction. This would allow broad activation of AMPK by many types of viruses internalized by such routes and provide a rapid response to restrict virus infection by inhibiting fatty acid synthesis.

Since AMPK activators are currently in the clinic to treat metabolic disorders such as type II diabetes [87], and restrict RVFV and KUNV replication in cell culture, they may prove to be useful antiviral therapeutics. Several AMPK activating drugs have been shown to reduce morbidity and mortality during lethal influenza infection in mice [88]. In addition, treatment of AMPK-activating drugs inhibited infection of HCMV and HIV in cells, and the addition of AMPK-activating drugs such as Metformin to current HCV treatment regimens had promising, albeit modest, effects on reducing patient viral loads [84], [89]–[92]. Infections with HCMV, HIV, and HCV have also been shown to inhibit AMPK activity [56], [84], [89], [92]. AMPK may have multiple effects on these infections since different downstream mechanisms have been implicated [56], [84], [89], [92], [93]; however, this suggests the possibility that some viruses have developed mechanisms of immune evasion that target AMPK. Taken together, AMPK plays a broad role in cellular innate immunity through potent inhibition of fatty acid synthesis, which is broadly utilized by viruses, suggesting that AMPK and perhaps other modulators of lipid biosynthesis are potential targets for broad pan-antiviral therapeutics.

## Materials and Methods

### Cells, Antibodies, Reagents, and Viruses

MEFs, BHK and U2OS cells were maintained at 37°C in DMEM supplemented with 10% FBS (Sigma), 100 µg/ml penicillin/streptomycin, 2 mM L-glutamine, and 10 mM Hepes. LKB1^−/−^ MEFs [94] were complemented with MIGR (Vector) or FLAG-LKB1-MIGR (LKB1 cDNA) retrovirus and sorted on GFP+ cells by FACS as previously described [40]. Rift Valley fever virus MP-12 was grown in Vero-E6 cells supplemented with 10% FBS [51]. RVFV was UV-inactivated in a Stratalinker. KUNV (gift from M. Diamond) was grown in BHK cells. VSV-GFP [95] was grown in BHK cells as described [96]. SINV-GFP virus [97] was grown in C636 cells [98]. All viruses were tittered by plaque assay in BHK cells. Antibodies were obtained from the following sources: anti-RVFV ID8 (gift from C. Schmaljohn USAMRIID), anti-KUNV 9NS1 (gift from R. Doms), anti-tubulin (Sigma), and anti-P-AMPK, t-AMPK, P-ACC, t-ACC, P-eEF2, t-eEF2 (Cell Signaling Technology). Fluorescently labeled secondary antibodies and BODIPY-TR were obtained from Invitrogen. HRP-conjugated antibodies were obtained from Amersham. A769662 was obtained from Santa Cruz. Other chemicals were obtained from Sigma.

### Plaque Assay

Viruses were plaqued on MEFs as indicated. Confluent monolayers were treated with serial dilutions of virus for two hours, after which the viral inoculums were removed, and cells were overlayed with 0.75% agarose in MEM, and incubated at 37°C for 48 hours. Cells were fixed in 10% formaldehyde, and stained with crystal violet. Plaque number was determined manually, and plaque diameter was measured using MetaXpress software and used to calculate areas.

### Viral Infections and Immunofluorescence

For all infections, washes and media changes were performed in the control untreated wells, as well as those infected with virus. Viral immunofluorescence experiments were performed in 96 well plates as previously described [99]. Briefly, cells were grown overnight in 96 wells plates, media was removed and fresh media was added. When appropriate, drug was added at the indicated concentration in 5 µl PBS, and cells were incubated at 37°C for 1 hour before addition of virus. Cells were infected with the indicated MOI of virus in complete media and spinoculated for 1 hour at 1200 RPM, and incubated at 37°C. Cells were fixed and processed for immunofluorescence as previously described 10 hours post infection for RVFV, SINV, and VSV, and 24 hours post infection for KUNV unless otherwise indicated [100]. Briefly, cells were fixed in 4% formaldehyde/PBS, washed twice in PBS/0.1% TritonX-100 (PBST), and blocked in 2% BSA/PBST. Primary antibodies were diluted in block, added to cells, and incubated overnight at 4°C. RVFV was stained with anti-RVFV ID8; KUNV was stained with anti-KUNV 9NS1. VSV and SINV expressed GFP, and did not require antibody staining. Cells were washed three times in PBST, and incubated in secondary antibody with Hoescht33342 (Sigma) counterstain for one hour at room temperature. Plates were imaged at 10× using an automated microscope (ImageXpress Micro), capturing four images per well per wavelength, and quantification was performed using MetaXpress image analysis software. Significance was determined using a Student's T-test. For immunofluorescence assays, a minimum of three wells per condition was imaged, with four images taken per well. To control for variability in baseline level of infection, a Student's T-test was performed on both the raw percent infection data in each individual experiment, and across a minimum of three replicate experiments where the untreated control had been normalized. Significance was determined if p<0.05 in all tests.

### One Step Growth Curve

MEFs were infected with RVFV MOI 1 in 6 well dishes and incubated at 37°C. Two hours post infection, inoculums was removed, and fresh medium was added. At indicated time point, medium was removed from infected cells and tittered on BHK cells by plaque assay.

### RVFV Binding Assay

MEFs were grown overnight in a 6 well dish. Medium was replaced with 1 mL of fresh complete medium and cells were chilled to 4°C for 10 minutes. RVFV (MOI 10) was added on ice, and cells were incubated at 4°C for 1 hour to allow virus binding. Cells were washed in PBS, then treated with either PBS or 0.25% trypsin to remove bound virus as previously described [101]. Cells were pelleted, then washed again, and lysed in Trizol to extract total RNA. Samples were then prepared for quantitative RT-PCR. cDNA was prepared from total RNA using M-MLV reverse transcriptase (Invitrogen) random primers, and transcripts were amplified by quantitative PCR. ΔΔCT was calculated for RVFV S segment using GAPDH as a cellular loading control.

### Time of Addition Assay

Time of addition experiments were performed as previously described [51]. U2OS cells were grown overnight, and the media was replaced. Cells were infected with RVFV (MOI 1), spun at 1200 rpm for 1 hour, and subsequently incubated at 37°C. 12 mM 2DG, 200 µM A769662, or 12 mM Ammonium Chloride were added either 1 hour prior to infection (−1), with infection (0), or 1, 2 or 4 hours after infection. 10 hours post infection cells were fixed in 4% formaldehyde in PBS and processed for immunofluorescence. Significance was determined using a Student's T test.

### Immunoblotting and Northern blotting

MEFs were infected with RVFV MOI 1 in 6 well dishes (∼50% infection) and incubated at 37°C for indicated time point. For protein analysis, cells were washed briefly in cold PBS and lysed in NP40 lysis buffer supplemented with protease (Boehringer) and phosphatase (Sigma) inhibitor cocktails. Samples were separated by SDS-PAGE and blotted as described [69]. HRP-conjugated secondary antibodies and Western Lightening Chemiluminescence Reagent were used for visualization. To analyze downstream effectors of AMPK, MEFs were treated with 12 mM 2DG, 10 µM oligomycin, or 100 uM A769662 for 4 hours, lysed and blotted as above.

For RNA analysis, cells were lysed in Trizol buffer, and RNA was purified as previously described [100]. To detect viral mRNA, total RNA from infected cells was separated on a 1% agarose/formaldehyde gel and blotted with the indicated probes as previously described [100]. Samples were quantified and normalized against controls using ImageQuant software.

### Cellular Lipid Staining

Cellular lipids were stained as previously described [82], [84]. MEFs were grown to confluence overnight, and then treated with PBS vehicle or 100 µM A769662 for 10 hours. Cells were fixed in 4% formaldehyde for 10 minutes and washed three times in PBS. Staining was performed with 10 µg/ml BODIPY-TR and counterstained with Hoescht33342 in 100 mM glycine in PBS overnight. Cells were washed three times in PBS and imaged using the ImageXpress Micro automated microscope. Integrated intensity of BODIPY signal per cell area was calculated using MetaXpress image analysis software. Significance was determined using a Student's T test.

### Fatty Acid Synthesis Bypass Assay

Exogenous palmitate addition was performed as previously described [102]. Delipidated Fetal Calf Serum and Albumin-bound palmitate were prepared as described [102] and obtained as a kind gift from Robert Rawson. U2OS cells were set up on day 0 in 96 well plates and grown over night in normal growth medium. On day 1 medium was removed and cells were washed briefly in PBS. Cells were treated with low glucose DMEM supplemented with 5% delipidated Fetal Calf Serum with or without 100 µM Albumin-bound palmitate, and incubated overnight. On day 2 cells were treated with 100 µM A769662 or PBS vehicle for 1 hour, and infected with RVFV for 10 hours. Cells were fixed, processed for immunofluorescence, and imaged at 10× using the automated microscope ImageXpress Micro, as described above. Quantification was performed using MetaXpress image analysis software. Significance was determined using a Student's T-test.

## Supporting Information

Figure S1 AMPK restricts RVFV. **A.** Time course of RVFV infection in WT and AMPKα1/AMPKα2^−/−^ MEFs. Cells were infected with RVFV and fixed at indicated time post infection. (RVFV, green; nuclei, blue) **B.** Quantification of **A.** A representative of triplicate experiments is shown.
(TIF)

Figure S2 AMPK inhibition leads to increased RVFV infection. **A.** U2OS cells were pretreated with 10 µM Compound C or PBS (untreated) for 1 hour and infected with serial dilutions of RVFV for 10 hours and processed for immunofluorescence. Data are displayed as the average percent infection relative to untreated control ± SD from triplicate experiments. * indicates p<0.05. **B.** Cellular Toxicity in response to drug treatment. U2OS were pretreated with 10 mM 2DG, 10 µM oligomycin, 100 µM A769662, 10 µM Compound C, 10 µg/ml STO609 or PBS (untreated) for 1 hour, infected with RVFV, and processed for immunofluorescence 10 hpi. Cell nuclei were counted using automated microscopy as a measure of cytotoxicity. Data are displayed as the average number of nuclei relative the untreated control ± SD from triplicate experiments.
(TIF)

Figure S3 Dose-dependent inhibition of RVFV infection.. U2OS cells were pretreated with serial dilutions of A769662 (**A**), 2DG (**B**), or STO609 (**C**) prior to infection with RVFV (MOI 1), and processed for immunofluorescence 10 hpi. Data are displayed as the average percent infection relative to the 0 drug control ± SD from triplicate experiments. * indicates p<0.05.
(TIF)

Figure S4 A769662 activates AMPK to restrict infection. **A.** WT and AMPKα1/AMPKα2^−/−^ MEFs were pretreated with 100 µM A769662 or PBS (untreated) for 1 hour, then infected with RVFV (MOI 1) for 10 hours and processed for immunofluorescence. Data are displayed as the average percent infection relative to the WT untreated control ± SD from triplicate experiments. * indicates p<0.05. **B.** Cell numbers from (**A**) as a measure of cell toxicity. Data are displayed as the average number of nuclei relative to the untreated ± SD from triplicate experiments.
(TIF)

Figure S5 Cellular ATP content is unchanged during RVFV infection. WT MEFs were treated with 2DG (12 mM), A769662 (µM), or infected with RVFV at MOI 2.5 or 12, spun at 1200 rpm for 1 hour, and incubated for 4 hours. ATP concentration was measured by luminescence. Data are displayed as average RLU relative to untreated control ±SD from triplicate experiments. * indicates p<0.05.
(TIF)

Figure S6 AMPK's role in the type I interferon response. **A–B.** WT and AMPKα1/AMPKα2^−/−^ MEFs were infected with RVFV for 10 hours. Expression of IFNβ (**A**) and OAS1 (**B**) were measured by qRT-PCR. Data are representatives of duplicate experiments. **C.** WT MEFs were treated with IFNβ for 15 minutes or 4 hours, lysed, and assayed by immunoblot for phospho-AMPK and phospho-ACC. Total AMPK and tubulin were assayed. A representative of triplicate experiments is shown. **D.** Quantification of **C.** using Image J software.
(TIF)

Figure S7 Quantification of Immunoblots using Image J software. **A–D.** Phosphorylation of AMPK and downstream effectors upon RVFV infection. WT MEFs were infected with RVFV (MOI 1) for 4 or 8 hours. Lysates were collected, assayed by immunoblot and quantified for phospho-AMPK (**A**), phospho-ACC2 (**B**), phospho-ACC1 (**C**), and phospho-eEF2 (**D**) normalizing to the tubulin loading control. Data are displayed as the average density relative to untreated at 4 hours from triplicate experiments. **E–H.** Phosphorylation of AMPK and downstream effectors in WT and AMPKα1/AMPKα2^−/−^ MEFs. Cells were treated with AMPK activators 2DG (12 mM), oligomycin (OM, 10 µM), and A769662 (100 µM) for 4 hours. Lysates were collected, assayed by immunoblot, and quantified as above for phospho-AMPK (**E**), phospho-ACC2 (**F**), phospho-ACC1 (**G**), and phospho-eEF2 (**H**) normalized to the tubulin loading control. Data are displayed as the average density relative to untreated at 4 hours from triplicate experiments.
(TIF)

Figure S8 UV-inactivated RVFV is replication incompetent. U2OS cells were infected with live (MOI 1) and UV-inactivated virus (equivalent volume to MOI 1) for 10 hours, and processed for immunofluorescence. (RVFV-N, green; nuclei, blue)
(TIF)

Figure S9 AMPK is not activated by RVFV in LKB1 null MEFs.LKB1−/−;LKB1 and LKB1−/−;Vec MEFs were infected with RVFV (MOI 1) for 4 hours. Lysates were collected and assayed by immunoblot for phospho-AMPK. Total AMPK and tubulin were assayed. Representative blot of duplicate experiments is shown.
(TIF)

Figure S10 **A:** mTORC1 is not required for AMPK-mediated restriction of RVFV. WT and AMPKα1/AMPKα2^−/−^ MEFs were pretreated with 10 nM Rapamycin or PBS for 1 hour and infected with RVFV (MOI 1) for 10 hours and processed for immunofluorescence. A representative of duplicate experiments is shown. **B.** Autophagy does not restrict RVFV. RVFV was plaqued in MEFs expressing a control hairpin RNA or a hairpin against Atg5. **C.** Atg5 mRNA expression by qRT-PCR in MEFs expressing a control hairpin RNA or a hairpin against Atg5 normalized to GAPDH.
(TIF)

Figure S11 Palmitate treatment does not inhibit AMPK activation or signaling. U2OS cells were treated with palmitate overnight, then treated with 2DG (12 mM) and A769662 (100 µM) for 10 hours. Lysates were collected and assayed by immunoblot for phospho-AMPK, and phospho-ACC. Total AMPK, ACC and tubulin were assayed. Representative blot of duplicate experiments is shown.
(TIF)

Figure S12 Addition of palmitate partially restores KUNV infection in the presence of A769662. **A.** U2OS cells were pretreated with 100 µM palmitate and 100 µM A769662 or PBS 1 hour prior to infection with KUNV (MOI 1). Cells were incubated for 16 hours, and processed for immunofluorescence. (KUNV-Ns1, green; nuclei, blue) **B.** Quantification of **A.** Data are displayed as the normalized percent infection relative to the non-A769662 treated control ±SD in triplicate experiments; * indicates p<0.05. **C.** Quantification of non-drug treated samples in (**A**). Palmitate treatment inhibited KUNV infection. Data are displayed as the normalized percent infection relative to the untreated vehicle control ±SD in triplicate experiments; * indicates p<0.05.
(TIF)

Text S1 The supporting information contains the methods used in Figures S1, S2, S3, S4, S5, S6, S7, S8, S9, S10, S11, S12.
(PDF)
