# Supplementary material for: AMP-Activated Kinase Restricts Rift Valley Fever Virus Infection by Inhibiting Fatty Acid Synthesis
Source: PLoS Pathog. 2012 Apr 19;8(4):e1002661. doi: 10.1371/journal.ppat.1002661 (PMC3330235; doi:10.1371/journal.ppat.1002661)
Supplement: Text S1 — The supporting information contains the methods used in Figures S1, S2, S3, S4, S5, S6, S7, S8, S9, S10, S11, S12. (PDF) [file ppat.1002661.s013.pdf]

AMP-ACTIVATED KINASE RESTRICTS RIFT VALLEY FEVER VIRUS INFECTION BY INHIBITING FATTY ACID SYNTHESIS

Theresa S. Moser, Daniel Schieffer, and Sara Cherry*

Supplementary Materials and Methods

ATP Assay

MEFs were infected with RVFV in white 96 well plates at MOI 2.5 or 12 (to infect 50 or 100% of cells, respectively), or treated with 12mM 2DG or 100 uM A769662, spun for 1 hour, and incubated at 37°C for 4 hours. ATP content was measured by luminescence with Cell Titer Glo reagent (Promega) according to the manufacturer's instructions.

IFN Assay

MEFs in 6 well dishes were treated with 10 U/ml interferon beta overnight, then infected with RVFV MOI 1 (sufficient for 50% infection) for 10 hours. Cells were lysed in Trizol and total RNA was extracted. Samples were then prepared for quantitative RT-PCR. cDNA was prepared from total RNA using M-MLV reverse transcriptase (Invitrogen) random primers, and transcripts were amplified by quantitative PCR. ΔΔCT was calculated for IFNβ and OAS1 using GAPDH as a cellular loading control.

Immunoblotting

For IFNβ experiments, cells were treated with 10 U/ml IFNβ for 15 minutes or 4 hours. For palmitate assays, medium was removed and cells were washed briefly in PBS. Cells were treated with low glucose DMEM supplemented with 5% delipidated Fetal Calf Serum with or without 100 µM Albumin-bound palmitate, and incubated 24 hours to mimic RVFV infection. Cells were washed briefly in cold PBS and lysed in NP40 lysis buffer supplemented with protease (Boehringer) and phosphatase (Sigma) inhibitor cocktails. Samples were separated by SDS-PAGE and blotted as described [69]. HRP-conjugated secondary antibodies and Western Lightening Chemiluminescence Reagent were used for visualization. Bands were quantified using Image J software, and normalized to the tubulin loading control.

Fatty Acid Synthesis Bypass Assay

U2OS cells were grown over night in normal growth medium. Medium was removed and cells were washed briefly in PBS. Cells were pretreated with low glucose DMEM supplemented with 5% delipidated Fetal Calf Serum with or without 100 µM Albumin-bound palmitate and 100uM A769662 1 hour prior to infection with KUNV (MOI 1). Cells were incubated for 16 hours, fixed, processed for immunofluorescence, and imaged at 10 X using the automated microscope ImageXpress Micro. Quantification was performed using MetaXpress image analysis software. Significance was determined using a Student's T-test.

Supplementary Figure Legends

Supplementary Figure 1: **A.** Time course of RVFV infection in WT and AMPKα1/AMPKα2$^{-/-}$ MEFs. Cells were infected with RVFV and fixed at indicated time post infection. (RVFV, green; nuclei, blue) **B.** Quantification of **A**. A representative of triplicate experiments is shown.

Supplementary Figure 2: **A.** U2OS cells were pretreated with 10 µM Compound C or PBS (untreated) for 1 hour and infected with serial dilutions of RVFV for 10 hours and processed for immunofluorescence. Data are displayed as the average percent infection relative to untreated control ± SD from triplicate experiments. * indicates $p<0.05$. **B.** Cellular Toxicity in response to drug treatment. U2OS were pretreated with 10 mM 2DG, 10 µM oligomycin, 100 µM A769662, 10 µM Compound C, 10 µg/ml STO609 or PBS (untreated) for 1 hour, infected with RVFV, and processed for immunofluorescence 10 hpi. Cell nuclei were counted using automated microscopy as a measure of cytotoxicity. Data are displayed as the average number of nuclei relative the untreated control ± SD from triplicate experiments.

Supplementary Figure 3: Dose response curves for drug treatment. U2OS cells were pretreated with serial dilutions of A769662 **(A)**, 2DG **(B)**, or STO609 **(C)** prior to infection with RVFV (MOI 1), and processed for immunofluorescence 10 hpi. Data are displayed as the average percent infection relative to the 0 drug control ± SD from triplicate experiments. * indicates $p<0.05$.

Supplementary Figure 4: **A.** WT and AMPKα1/AMPKα2$^{-/-}$ MEFs were pretreated with 100 µM A769662 or PBS (untreated) for 1 hour, then infected with RVFV (MOI 1) for 10 hours and processed for immunofluorescence. Data are displayed as the average percent infection relative to the WT untreated control ± SD from triplicate experiments. * indicates $p<0.05$. **B.** Cell numbers from **(A)** as a measure of cell toxicity. Data are displayed as the average number of nuclei relative to the untreated ± SD from triplicate experiments.

Supplementary Figure 5: Cellular ATP content in response to RVFV infection. WT MEFs were treated with 2DG (12 mM), A769662 (μM), or infected with RVFV at MOI 2.5 or 12, spun at 1200 rpm for 1 hour, and incubated for 4 hours. ATP concentration was measured by luminescence. Data are displayed as average RLU relative to untreated control ±SD from triplicate experiments. * indicates $p<0.05$.

Supplementary Figure 6: AMPK's role in the type I interferon response. **A-B.** WT and AMPKα1/AMPKα2$^{-/-}$ MEFs were infected with RVFV for 10 hours. Expression of IFNβ **(A)** and OAS1 **(B)** were measured by qRT-PCR. Data are representatives of duplicate experiments. **C.** WT MEFs were treated with IFNβ for 15 minutes or 4 hours, lysed, and assayed by immunoblot for phospho-AMPK and phospho-ACC. Total AMPK and tubulin were assayed. A representative of triplicate experiments is shown. **D.** Quantification of **C.** using Image J software.

Supplementary Figure 7: Quantification of Immunoblots using Image J software. **A-D.** Phosphorylation of AMPK and downstream effectors upon RVFV infection. WT MEFs were infected with RVFV (MOI 1) for 4 or 8 hours. Lysates were collected, assayed by immunoblot and quantified for phospho-AMPK **(A)**, phospho-ACC2 **(B)**, phospho-ACC1 **(C)**, and phospho-eEF2 **(D)** normalizing to the tubulin loading control. Data are displayed as the average density relative to untreated at 4 hours from triplicate experiments. **E-H.** Phosphorylation of AMPK and downstream effectors in WT and AMPKα1/AMPKα2$^{-/-}$ MEFs. Cells were treated with AMPK activators 2DG (12 mM), oligomycin (OM, 10 μM), and A769662 (100 μM) for 4 hours. Lysates were collected, assayed by immunoblot, and quantified as above for phospho-AMPK **(E)**, phospho-ACC2 **(F)**, phospho-ACC1 **(G)**, and phospho-eEF2 **(H)** normalized to the tubulin loading

control. Data are displayed as the average density relative to untreated at 4 hours from triplicate experiments.

Supplementary Figure 8: UV-inactivated RVFV is replication incompetent. U2OS cells were infected with live (MOI 1) and UV-inactivated virus (equivalent volume to MOI 1) for 10 hours, and processed for immunofluorescence. (RVFV-N, green; nuclei, blue)

Supplementary Figure 9: AMPK activation in LKB1 null MEFs.LKB1 -/-;LKB1 and LKB1-/-;Vec MEFs were infected with RVFV (MOI 1) for 4 hours. Lysates were collected and assayed by immunoblot for phospho-AMPK. Total AMPK and tubulin were assayed. Representative blot of duplicate experiments is shown.

Supplementary Figure 10: A: mTORC1 is not required for AMPK-mediated restriction of RVFV. WT and AMPKα1/AMPKα2 $^{-/-}$ MEFs were pretreated with 10 nM Rapamycin or PBS for 1 hour and infected with RVFV (MOI 1) for 10 hours and processed for immunofluorescence. A representative of duplicate experiments is shown. **B.** Autophagy does not restrict RVFV. RVFV was plaqued in MEFs expressing a control hairpin RNA or a hairpin against Atg5. **C.** Atg5 mRNA expression by qRT-PCR in MEFs expressing a control hairpin RNA or a hairpin against Atg5 normalized to GAPDH.

Supplementary Figure 11: Palmitate treatment did not inhibit AMPK activation or signaling. U2OS cells were treated with palmitate overnight, then treated with 2DG (12 mM) and A769662 (100 μM) for 10 hours. Lysates were collected and assayed by immunoblot for phospho-AMPK, and phospho-ACC. Total AMPK, ACC and tubulin were assayed. Representative blot of duplicate experiments is shown.

Supplementary Figure 12: Addition of palmitate partially restores KUNV infection in the presence of A769662. **A.** U2OS cells were pretreated with 100 µM palmitate and 100 µM A769662 or PBS 1 hour prior to infection with KUNV (MOI 1). Cells were incubated for 16 hours, and processed for immunofluorescence. (KUNV-Ns1, green; nuclei, blue) **B.** Quantification of **A**. Data are displayed as the normalized percent infection relative to the non-A769662 treated control ±SD in triplicate experiments; * indicates $p<0.05$. **C.** Quantification of non-drug treated samples in (**A**). Palmitate treatment inhibited KUNV infection. Data are displayed as the normalized percent infection relative to the untreated vehicle control ±SD in triplicate experiments; * indicates $p<0.05$.
